# The Indigestibility of Certain Articles

**Published:** 1892-10

**Authors:** 


### SELECTIONS.

### The Indigestibility of Certain Articles.

That there are certain articles of food which are indigestible mainly on account of chemical incompatibility with the fluids of digestion, must be accepted as true. However, a very much larger number of articles are indigestible mainly because they are tough, rather than either soft or friable, and hence are not well adapted to the requirement of being masticated to a pulp before being swallowed. Let us consider a few examples, which may be taken as sufficient to illustrate the entire class.

An egg is indigestible if boiled until the albumen is solidly coagulated. It is quite easy of digestion if eaten raw, soft boiled, or boiled several hours until it becomes mealy. The ordinary hard-boiled egg is usually swallowed in lumps which expose but little of their surface to the action of the gastric juice. Hence, its digestion is very slow, indeed.

Mashed potatoes, or old potatoes which boil mealy are easy of digestion, while new potatoes, which are almost invariably swallowed in chunks have a bad reputation. So, also, fried potatoes, in which the potato is well protected by a coating of grease, bid defiance to the digestive fluids; while mashed potatoes and gravy or butter gives no especial trouble.

Other articles that may be mentioned are green cucumbers, green corn, watermelon preserves, cheeese, "sad" bread, cake or dumplings, pie-crust (especially the well-soaked under-crust), and many others. Gladstone is reported to have said that he gives each mouthful of food thirty-two bites in masticating. Our own rule is to continue chewing a mouthful of food as long as it gives pleasure to the taste. At any rate, it may be laid down as a rule, that very seldom will food prove indigestible, if the one who eats it is thoroughly conscientious in reducing every bit of it to a fine pulp before swallowing it. Our reply to patients when asked what food is allowed, is generally, "Anything in reasonable quantity, that you will chew thoroughly," having due regard, of course, to special diseased conditions and circumstances.

Following the principles outlined above, persons of no more than the average digestive power may safely indulge in a meal composed of the indigestible articles mentioned herein.—*The Times and Register.*

